# Investigating the role of SPECT/CT in dynamic sentinel lymph node biopsy for penile cancers

**DOI:** 10.1007/s00259-017-3636-1

**Published:** 2017-02-07

**Authors:** Ziauddin Zia Saad, Savvas Omorphos, Sofia Michopoulou, Svetislav Gacinovic, Peter Malone, Raj Nigam, Asif Muneer, Jamshed Bomanji

**Affiliations:** 1 Department of Nuclear Medicine, University College Hospitals London, London, UK; 2 Institute of Nuclear Medicine, 5th Floor,, UCLH NHS Foundation Trust, 235 Euston Road, London, NW1 2BU UK; 3 Department of Urology, University College Hospitals London, London, UK

**Keywords:** Planar lymphoscintigraphy, SPECT/CT lymphoscintigraphy, Drainage basin, Sentinel node, Cancer of the penis

## Abstract

**Purpose:**

Currently, most centres use 2-D planar lymphoscintigraphy when performing dynamic sentinel lymph node biopsy in penile cancer patients with clinically impalpable inguinal nodes. This study aimed to investigate the role of SPECT/CT following 2-D planar lymphoscintigraphy (dynamic and static) in the detection and localization of sentinel lymph nodes in the groin.

**Methods:**

A qualitative (visual) review was performed on planar followed by SPECT/CT lymphoscintigraphy in 115 consecutive patients (age 28–86 years) who underwent injection of ^99m^Tc-nanocolloid followed by immediate acquisition of dynamic (20 min) and early static scans (5 min) initially and further delayed static (5 min) images at 120 min followed by SPECT/CT imaging. The lymph nodes detected in each groin on planar lymphoscintigraphy and SPECT/CT were compared.

**Results:**

A total of 440 and 467 nodes were identified on planar scintigraphy and SPECT/CT, respectively. Overall, SPECT/CT confirmed the findings of planar imaging in 28/115 cases (24%). In the remaining 87 cases (76%), gross discrepancies were observed between planar and SPECT/CT images. SPECT/CT identified 17 instances of skin contamination (16 patients, 13%) and 36 instances of in-transit lymphatic tract activity (24 patients, 20%) that had been interpreted as tracer-avid lymph nodes on planar imaging. In addition, SPECT/CT identified 53 tracer-avid nodes in 48 patients (42%) that were not visualized on planar imaging and led to reclassification of the drainage basins (pelvic/inguinal) of 27 tracer-avid nodes.

**Conclusions:**

The addition of SPECT/CT improved the rate of detection of true tracer-avid lymph nodes and delineated their precise (3-D) anatomic localization in drainage basins.

## Introduction

Penile cancer is a rare malignancy in Western Europe and North America. In England, the incidence has increased by approximately 20% over the last 40 years [1], and there are now just over 500 cases annually in the United Kingdom. Penile cancer has a characteristic stepwise lymphatic dissemination pattern, first to the inguinal lymph nodes and then to the pelvic lymph nodes. Haematogenous spread usually occurs in advanced stages of disease [2].

The single most important prognostic indicator is the presence of metastatic disease in the inguinal lymph nodes. Metastatic disease in a single inguinal lymph node is associated with a 5-year survival rate of over 80%. However, if the number of metastatic inguinal lymph nodes exceeds two, the 5-year survival rate is reduced to 40% [3]. Since only 15–20% of clinically impalpable inguinal nodes (cN0) harbour metastatic disease, performing a radical inguinal lymphadenectomy in all of these patients would result in a high risk of overtreatment [4]. The concept of sentinel lymph node biopsy (SNB) was first coined by Cabanas in 1977 [5], labelling the first lymph node receiving the lymphatic drainage from the primary neoplastic site as the “sentinel lymph node”, and in the presence of lymphatic spread, this would be the first lymph node to host the metastatic disease. The original study by Cabanas used the superficial epigastric vein as an anatomical landmark, with the sentinel node always medial to this vein. However, the incorporation of dynamic studies by Horenblas et al. followed by correlation with SPECT/CT confirmed the individual variability in the drainage pattern of each tumour, indicating that the sentinel node could be located outside the superomedial zone [6]. Since that time, the concept of sentinel lymph node biopsy has been evaluated, accepted, and established in many clinical pathways [7]. Its application in penile cancer was first highlighted by a group at the Netherlands Cancer Institute in the 1990s, followed by several other groups [8–10]. The approach has offered a minimally invasive technique for identification of micro-metastases in patients with cN0 disease, thus reducing the need for radical inguinal lymphadenectomy.

The superiority of single-photon emission computed tomography (SPECT) and hybrid SPECT/CT technology over planar nuclear imaging has been demonstrated in a number of benign and malignant disease processes [11–15]. Despite this, there is still uncertainty with regard to the use and benefits of SPECT/CT in penile cancer. The aim of this study was to prospectively compare the use of conventional planar lymphoscintigraphy with SPECT/CT in patients undergoing preoperative imaging prior to dynamic sentinel lymph node biopsy.

## Materials and methods

A total of 115 patients underwent both planar lymphoscintigraphy and SPECT/CT, and the data sets were analysed visually to identify discrepancies in nodal detection between these imaging modalities. Nodes were classified as belonging to the left or right side and were localized to the inguinal or pelvic basin.

SPECT/CT was performed after conventional planar lymphoscintigraphy in order to establish whether this imaging modality identified additional lymph nodes, allowed better delineation of the position of the sentinel lymph nodes relative to adjacent structures, detected lymphatic channels falsely identified as the sentinel lymph node, and allowed assessment of mis-localization secondary to scatter. In addition, we explored whether any inadvertent radioactive contamination was detected by SPECT/CT while being either missed completely or identified as a positive lymph node on planar imaging.

### Study period

The study period was June 2011 to December 2015.

### Patient population

One hundred and nineteen (119) consecutive patients were included in the study; however, four patients were excluded, in two cases because of a mechanical defect in SPECT/CT and in two further cases owing to a delay in the SPECT/CT acquisition, as a result of which the SPECT/CT was not completed. Therefore, the final population for data analysis comprised 115 patients (all adults; age range 28–86; median age 56 years). The institutional review board approved the protocol as a standard of care and all patients were accepted for inclusion, as they had already undergone surgery for the primary lesion and had confirmed penile squamous cell carcinoma (stage ≥ T1G2) and cN0 disease.

### Patient preparation and administration of radiopharmaceutical

Patients attended the nuclear medicine department following application of local anaesthetic (EMLA^®^) to the area proximal to the site of tumour excision. Approximately 30 MBq of technetium-99 m nanocolloid (range 20–40 MBq) in 0.4 ml was injected intradermally just proximal to the surgical scar at four points, giving an effective radiation dose of between 0.35 and 0.45 mSv.

### Image sequence, acquisition techniques, and parameters

All images were acquired on a hybrid SPECT/CT system: a dual-head Infinia Hawkeye 4, Model H3000WL (General Electric Medical Systems, Milwaukee, WI, USA). The acquisitions were carried out using a low-energy high-resolution (LEHR) collimator with zoom of x1 and keeping the energy window(s) to 140 keV, 20% width, and 3% offset.

A strict image sequence was followed in all cases. The dynamic anterior acquisition was performed immediately after the radiotracer injection with dual-head projections of 120x10 s, 128x128 matrix, 180°/detector for a total of 20 min. This was immediately followed by a 5-min early static anterior image acquisition from the top of the thighs to the lower abdomen. After an interval of 2 h post-injection, the patient underwent a further 5-min static anterior (delayed) image acquisition, again from the top of the thighs to the lower abdomen. This was immediately followed by SPECT/CT acquisitions with a standard low-dose CT protocol of 140 kVp, 2.5 mA, 14-s rotations, and the full 40-cm scan range, covering an identical field of view to planar imaging.

### Data reconstruction, display and reporting, and probe localization

**Fig. 1:**
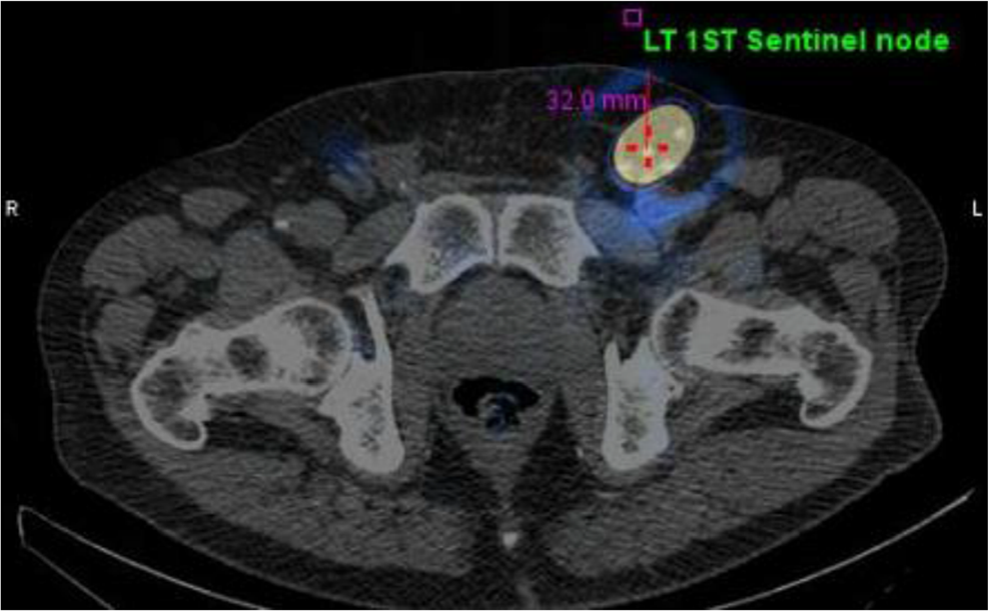
Generated image for urology colleagues to facilitate intraoperative sentinel node localization

The reconstruction and display was performed on a GE Xeleris workstation. The displayed image data were “electronically windowed” at the console to highlight the sentinel node(s) and drainage path(s). Both SPECT images and computed tomography images were matched and then reconstructed using GE Volumetric MI Evolution software. The images were reviewed by duty nuclear physicians, confirming the position of the sentinel node(s) before the skin was marked using a cobalt-57 pencil. The principle of detecting the first node seen on the dynamic images showing radioisotope uptake was followed in order to isolate the sentinel node, and was confirmed by early static image. In cases of non-visualized dynamic and early static images, the most intense tracer-avid inguinal basin node (on delayed static and SPECT/CT images) was perceived and called the sentinel node, and the others were highlighted as echelon nodes. The final screenshot images were generated for our urology colleagues, demonstrating the sentinel and echelon nodes with depth estimation (Fig. 1). Intraoperatively, all of the avid nodes were probe-localized, and these inguinal nodes were surgically excised for pathological analysis.

### Data analysis

As our study primarily focused on visual assessment of the images, the data were analysed qualitatively in two phases. During the first phase of image analysis, early and delayed static planar lymphoscintigraphy images were reviewed to determine the total number of visualized tracer-avid nodes, along with their localization to the inguinal and pelvic basins. The second phase involved comparing the delayed static images with SPECT/CT images.

We performed a detailed visual (qualitative) analysis in which the two data sets (2-D planar vs 3-D SPECT/CT) were compared by two senior nuclear physicians (JB and ZS). If there was no discrepancy between the delayed static and SPECT/CT images, the value of additional SPECT/CT was classified as insignificant or just confirmatory. On the other hand, if there were discrepancies—for example, with respect to nodal yield (increased or decreased number of tracer-avid nodes), anatomic localization of nodes to the designated inguinal and pelvic basins, retention of focal tracer activity in lymphatic tracts, or true nodal activity and other focal tracer-avid spots (e.g., contaminations)—then the value of SPECT/CT was classified as significant.

### Statistical analysis

Although we reported our results descriptively, we also performed McNemar’s test (PASW Statistics for Windows, version 18.0; SPSS Inc., Chicago, IL, 2009) using a continuity correction to evaluate the null hypothesis that the two modalities are equally good at detecting tracer-avid lymph nodes. The level of statistical significance was set at *p* = 0.05.

## Results

A total of 440 avid nodes were identified on delayed planar scintigraphy, with 234 on the right side and 206 on the left, of which 357 were perceived to be located in the inguinal basin and 83 located within the pelvis. A total of 467 avid nodes were identified on SPECT/CT images, with 253 on the right side and 214 on the left, of which 355 were noted to be located in the inguinal basin and 112 nodes in the pelvis (Table 1).

**Table 1:** Number of nodes identified using planar lymphoscintigraphy vs SPECT/CT and nodal distribution in the inguinal and pelvic basins and the right and left groin

No. of nodes	Total		Right groin		Left groin		Inguinal basin		Pelvic basin	
	Planar	SPECT/CT	Planar	SPECT/CT	Planar	SPECT/CT	Planar	SPECT/CT	Planar	SPECT/CT
Total	440	467	234	253	206	214	357	355	83	112
Average per patient	3.83	3.92	2.03	2.2	1.79	1.86	3.1	3.09	0.72	0.97
Standard deviation	2	1.93	1.36	1.36	1.34	1.24	1.79	1.48	0.93	0.92

Overall, SPECT/CT confirmed the findings of planar imaging in 28/115 cases (24%), and a total of 387 nodes were identified by both modalities (Table 2). In the aforementioned 28 patients, there was a perfect nodal match between planar and SPECT/CT images. In the remaining 87 cases (76%), gross discrepancies between planar and SPECT/CT images were observed with regard to the number of identified tracer-avid nodes, localization of nodes to the inguinal or pelvic drainage basin, retention of activity in lymphatic tracts, and contaminations resembling tracer-avid nodes.

**Table 2:** Contingency matrix of lymph node detection on SPECT/CT vs. planar scintigraphy

		SPECT/CT		
		Yes	No	Total – planar
Planar	Yes	387	53^b^	440
	No	80^a^	0	
	Total – SPECT	467		

^a^ Includes 27 tracer-avid nodes that were reclassified from the inguinal to the pelvic drainage basin or vice versa on the basis of SPECT/CT findings

^b^ Includes 17 instances of skin contamination and 36 instances of in-transit lymphatic tract activity

**Fig. 2:**
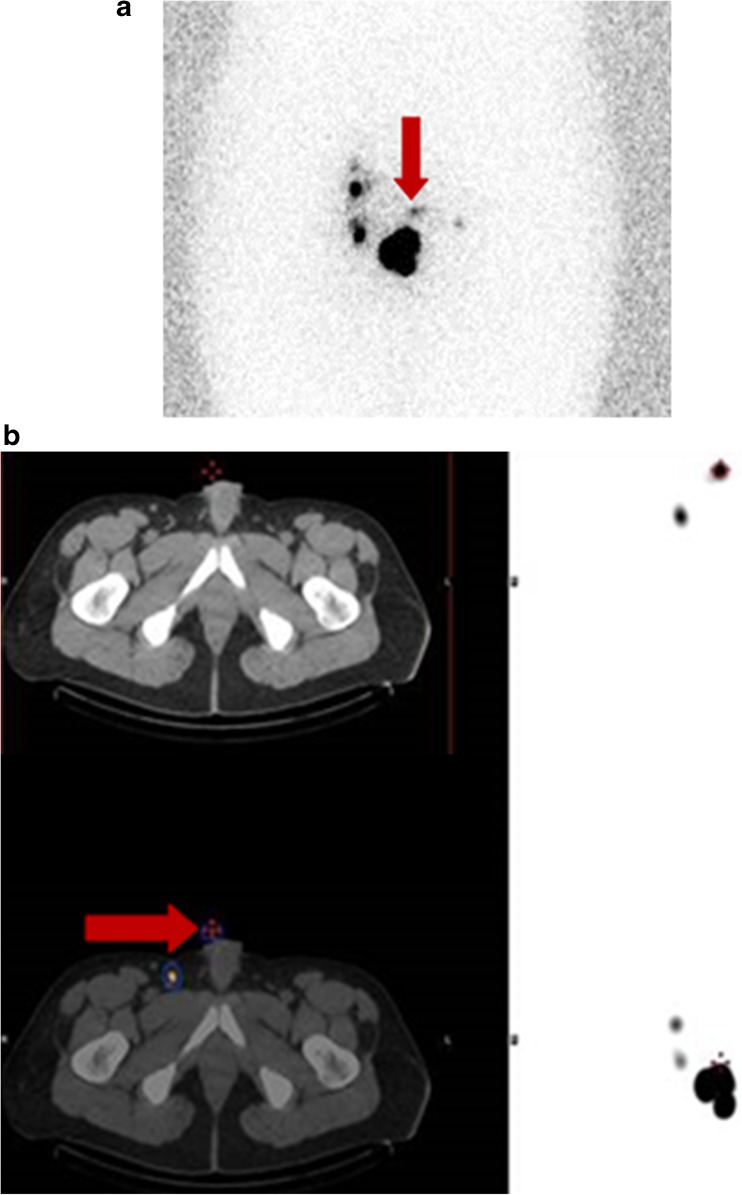
**a** Planar image showing tracer uptake (*red arrow*) resembling a lymph node in the suprapubic region. **b** SPECT/CT image demonstrates that the uptake noted in **a** actually represents contamination (*red arrow*)

**Fig. 3:**
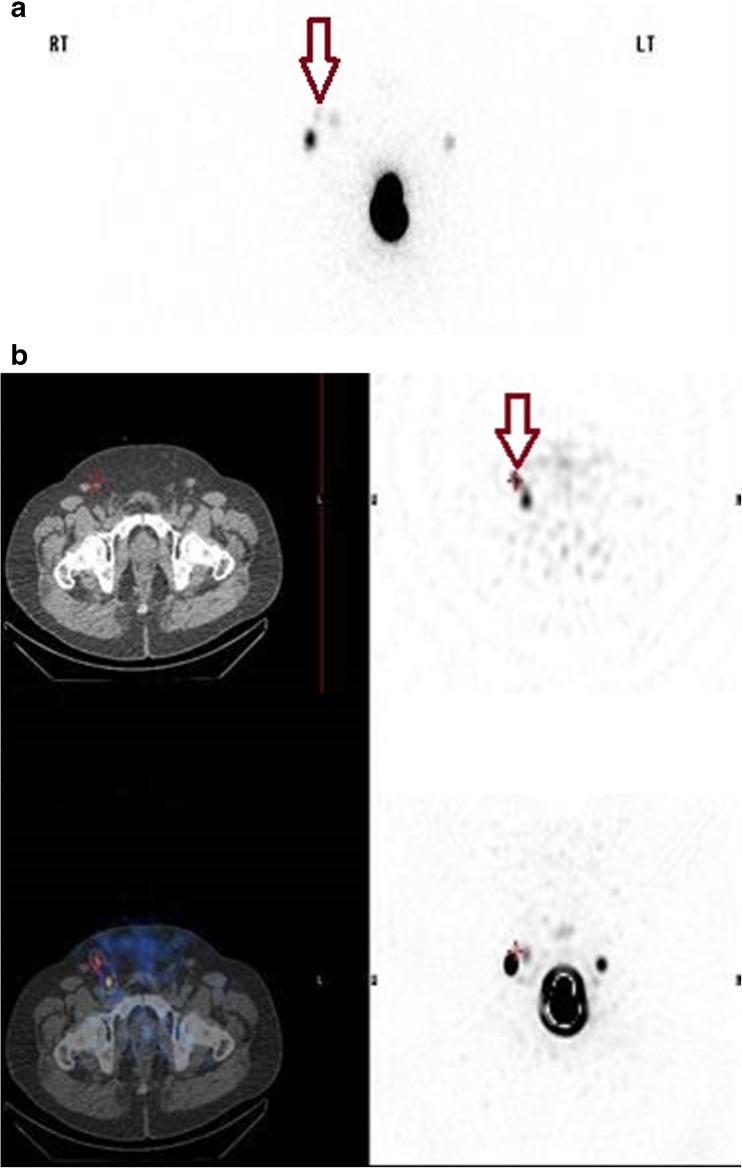
**a** Planar image shows at least three tracer-avid foci (maroon arrow) resembling lymph nodes in the right groin. **b** SPECT/CT images demonstrates that one of the foci in **a** is essentially an in-transit activity (maroon arrow), potentially contributing to false-positive rates

In the 76% of patients in whom gross discrepancies were observed between planar and SPECT/CT images, SPECT/CT identified 17 instances of skin contamination in 16 patients (13%) (Fig. 2a, b) and delineated 36 instances of in-transit lymphatic tract activity (Fig. 3a, b) in 24 patients (20%). These 53 cases were considered to represent tracer-avid lymph nodes on planar imaging and hence contributed to a significant false-positive rate. Furthermore, SPECT/CT led to a reclassification of 27 tracer-avid nodes (in nearly 20% of patients) from the inguinal basin to the pelvis or vice versa. In addition, 21 tracer-avid nodes in the inguinal basin and 32 tracer-avid nodes in the pelvis that were identified in 48 patients (42%) on SPECT/CT images were not visualized on conventional 2-D planar scintigraphy.

Although the difference in the absolute number of nodes between the two modalities was small when the images were analysed individually, in real-time clinical practice, the relative difference was substantial, as shown by the contingency matrix in Table 2. This was also demonstrated on McNemar’s test, which revealed statistically significant differences in node detection between planar scintigraphy and SPECT/CT, verifying the superiority of SPECT/CT in lymph node identification (two-tailed *p* = 0.024).

In the clinical scenario, bilateral inguinal nodes should be detected, reflecting the lymphatic drainage of the penis, unless surgery in the form of an inguinal lymphadenectomy has already been performed, in which case unilateral visualization is expected. In our cohort, unilateral drainage was found in 26 patients and bilateral drainage in the remaining 89 patients. In addition, it was observed that dynamic and early static images demonstrated tracer progression to a node (true sentinel node by definition) in 97 cases (84%). Furthermore, there were 18 cases (16%) where dynamic and early static images did not show nodal visualization in either groin. Tracer-avid nodes, however, were visualized in 112 cases (97%) at the end of the delayed static images at 120 min.

SPECT/CT images, on the other hand, showed at least one tracer-avid node in the inguinal basins either unilaterally or bilaterally in all 115 cases. Of the three cases which did not show tracer-avid nodes following delayed static images, SPECT/CT identified four inguinal basins with a cumulative nine nodes. More importantly, two of these additional four basins contained metastatic nodes.

## Discussion

Cross-sectional imaging such as X-ray computed tomography (CT) or magnetic resonance imaging (MRI) can detail structural changes, but is unable to supply precise information on the functional status of pathological lesions. On the other hand, scintigraphy yields functional data sets defining biochemical and metabolic changes, but suffers from the drawback that it is often difficult to precisely define the extent of disease, owing to the lack of anatomic definition. However, the co-registered images provided by hybrid SPECT/CT offer significant gains in observer confidence, surgical precision, and diagnostic accuracy [16].

Since the publication of penile cancer guidelines by the European Association of Urology (EAU) in 2001–2002 [17], the standard protocols have not acknowledged the routine use of SPECT/CT in sentinel lymph node biopsy. Our group is amongst the very few supra-regional penile cancer centres in the UK to routinely perform SPECT/CT in patients undergoing dynamic sentinel lymph node biopsy. From a surgical perspective, SPEC/CT identified the expected nodal yield on surgical exploration, which led to the modification of the surgical procedure so that all the nodes identified on SPEC/CT were localized in the inguinal basin using a gamma probe. It also helped to ensure that unnecessary explorations due to spillage or misregistration artefacts or activity from the tracer-avid pelvic nodes were not performed. Thus far, histological analysis has shown metastatic disease limited to the sentinel nodes rather than the echelon nodes, based on a pathological analysis which allows detection within 2-μm slice thickness.

We believe that such routine incorporation of SPECT/CT into the imaging protocol when performing dynamic sentinel lymph node biopsy in patients with penile cancer can further improve the sensitivity and specificity for the detection of involved occult lymph nodes, which are both around 90% when using ^99m^Tc-nanocolloid imaging and ultrasonography preoperatively in conjunction with intraoperative use of patent blue dye and a handheld gamma probe [18].

The dynamic and early planar images have been reported to visualize the first draining lymph node in approximately 85% of cases [19], and this was confirmed by the present study, in which dynamic and early static images enabled visualization in 84% of patients. By definition, the first lymph node(s) draining directly from the injection site is classified as the sentinel node. In the case of multiple visible nodes without visible afferent lymphatics, the first node appearing in a basin is considered to be the sentinel node. When there is non-visualization on dynamic and early and delayed planar imaging, it is generally accepted practice to continue to acquire delayed static images for up to 4 h until a tracer-avid node becomes visible. If a tracer-avid node has still not been visualized after 4 h, proceeding to intraoperative gamma probe localization is recommended. However, the experience in our cohort has demonstrated that inclusion of SPECT/CT, with its better contrast and resolution, enables convincing localization of tiny tracer-avid nodes that are otherwise difficult to detect, and therefore has the potential to obviate the need for further delayed static images (which may continue up to 4 h post-injection). A recent publication has addressed this issue in detail [20].

**Fig. 4:**
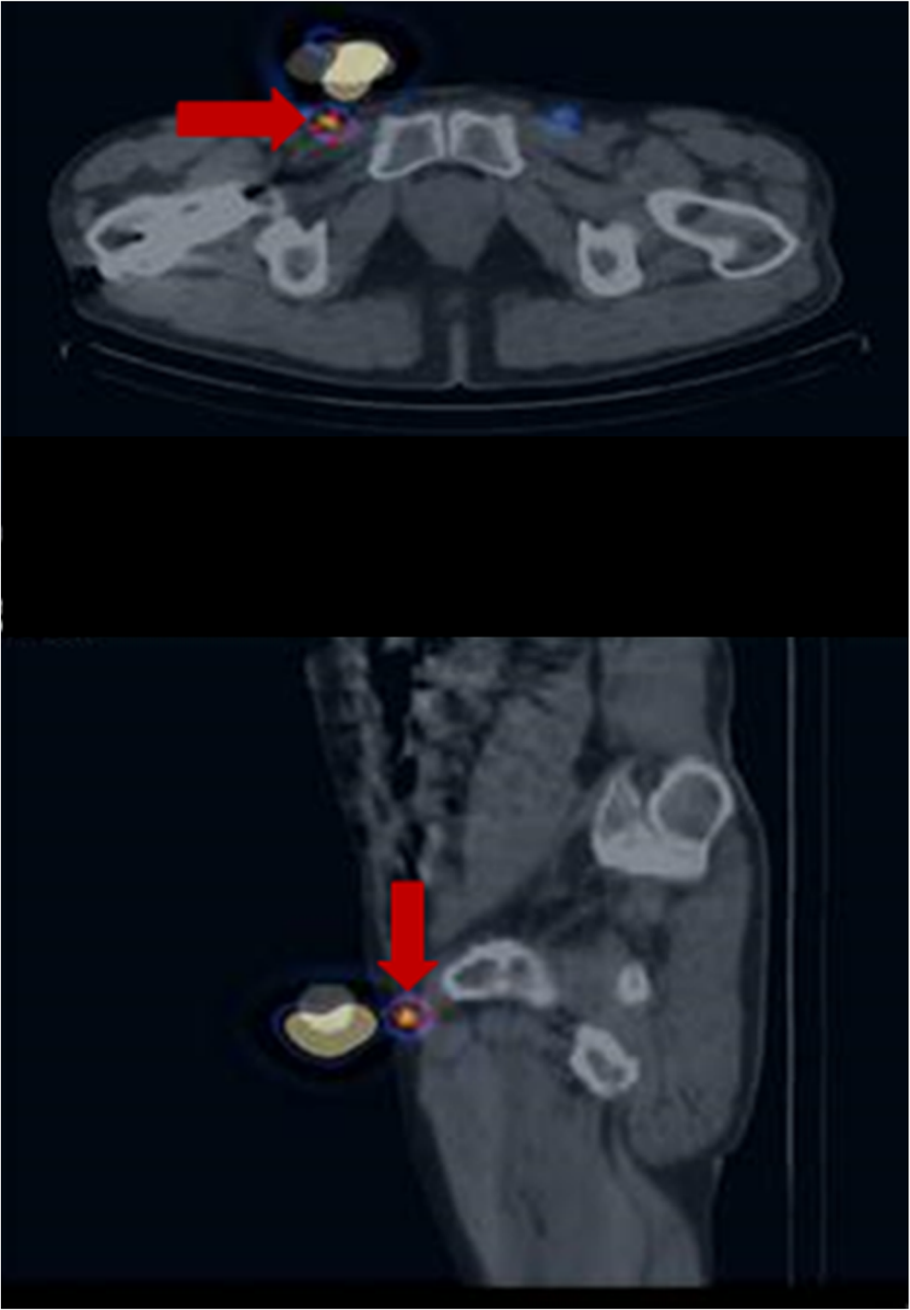
SPECT/CT clearly localizes a node (*red arrows*) that was missed on planar imaging due to adjacent intense activity

Similarly, 2-D planar lymphoscintigraphy has been found to accurately predict the number of nodes in only 81% of the basins, overlooking nodes that are superimposed and cannot be separated from other nodes or from the injection site (Fig. 4) or lymphatic channels, and nodes that are beyond the resolution of the planar images [21, 22]. This limitation was again highlighted in the present study, in which 53 additional nodes were detected using SPECT/CT. We attribute the improvement in detection accuracy with SPECT/CT to its tomography and ability to overcome the effects of the inadequacies of planar techniques, along with the use of non-standardized acquisition protocols.

Although the acquisition of SPECT/CT images increases the time required for preoperative imaging, it has the key advantage of being able to precisely localize sentinel lymph nodes for removal by the surgeon. The anatomical landmarks for surgeons mainly relate to the depth of the node and the proximity to the femoral vein and femoral canal, in addition to the differentiation between nodes in the inguinal and pelvic regions. This is not obvious on planar scanning, but is better visualized on SPECT/CT, enabling planning of the procedure, particularly in patients with a high BMI, when the nodes are located deep in the inguinal region and gamma probe activity is detected over a wider area.

The acquisition and correct interpretation of 3-D reconstructive SPECT/CT preoperative images can reduce the time spent exploring the groins for the lymph nodes. Overall, SPECT/CT offers a better detection rate than planar imaging, or even intraoperative gamma probe detection on its own [23, 24]. Two separate studies have also demonstrated the cost-effectiveness of using SPECT/CT in combination with planar lymphoscintigraphy for the detection of sentinel lymph nodes at complex sites [25, 26]. Common pitfalls in the interpretation of planar images are the localization of tracer-avid nodes to the incorrect drainage basin and detection of false-positive non-nodal sites of uptake due to scattering of radioactivity from the injection site. In the present study, SPECT/CT led to reclassification of 27 tracer-avid nodes from the inguinal to the pelvic drainage basin or vice versa on the basis of anatomical landmarks and compartments. It is important to mention here that the technique was not primarily used to visualize pelvic nodes, and these were not removed even if they were tracer-avid. SPECT/CT ensured that unnecessary exploration was not performed in cases where the planar imaging showed the presence of inguinal nodes (which were subsequently found to be located in the pelvis). Surgically, this resulted in a reduction in the surgical operative time, limited the dissection to the inguinal nodes, and reassured the surgeon that residual gamma probe activity corresponded to activity in the pelvic nodes or skin contamination.

An important prerequisite of intraoperative surgical removal of the lymph nodes is the use of a gamma probe. Therefore, the additional nodes on SPECT/CT would likely have been detected if planar scans with gamma probe activity were used, provided the surgeon had ensured that the probe detected background radiation at the end of the procedure. The study has demonstrated that the addition of SPECT/CT allows for better surgical planning so that the surgeon knows the expected nodal yield from each groin and can account for the additional gamma probe activity in cases of pelvic node radioisotope uptake or spillage. This study also demonstrates that SPECT/CT per se is adequate for the detection of the sentinel node and echelon nodes, such that patients can undergo a radioisotope injection followed by a 2-h SPEC/CT. This provided the surgical team with ample imaging data to explore the inguinal region and remove the relevant nodes.

**Fig. 5:**
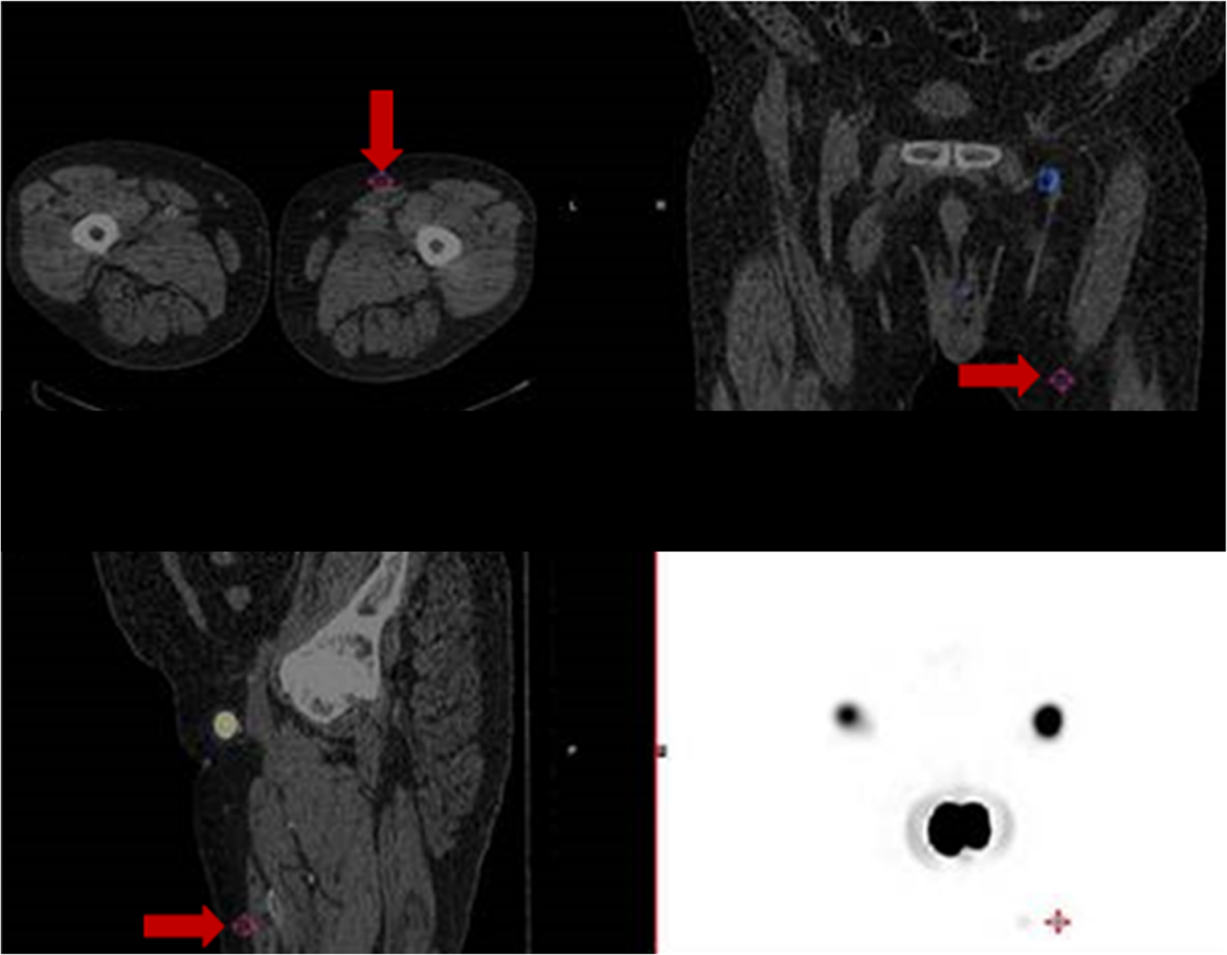
An unusual drainage pattern, with localization of a node to the left mid-thigh (*red arrows*)

This study also confirmed the consistent and stepwise lymphatic drainage which occurs in penile cancer, first to the inguinal and then to the pelvic basin; however, on SPECT/CT, we also noted a solitary node which escaped the usual pattern of drainage and was located at an unusual site (mid-thigh) (Fig. 5).

The main limitation of our study was having a conceptual agreement when the true sentinel node was not visualized at the end of dynamic imaging, and referring to the subsequently appearing node(s) at delayed time points as tracer-avid nodes which would have a high probability of being the sentinel node. It is evident from our study that SPECT/CT does not serve to replace planar lymphoscintigraphy, especially the dynamic component, which is still the gold standard for identification of the sentinel lymph node.

Another limitation that we observed was that although SPECT/CT could identify the nodes in the inguinal basin, it could not differentiate or predict which specific node would harbour metastatic disease, as the most important prognostic indicator for patients with penile cancer is the presence of metastatic disease in the inguinal lymph nodes. Currently, the additional lymph nodes may not be significant in terms of the detection of micro-metastatic disease histologically; however, it did provide reassurance that the lymph nodes draining the penis which may harbour micro-metastatic disease have all been removed. As the central focus of the current article was SPECT/CT imaging, and follow-up data for most of the patients in this cohort with regard to surgical and histopathological findings are still under evaluation, the correlative aspect between imaging results, surgery, and histopathological results was not specifically addressed.

This study has demonstrated that the use of SPECT/CT in conjunction with planar imaging offers numerous advantages, i.e., detection of false positives due to contamination and false negatives due to missed nodes on 2D planar imaging, more accurate localization of the drainage basin and region, detection of lymphatic tract leaks, identification of lymph nodes lying too close to the injection site for detection on planar scintigraphy, identification of sentinel nodes having little radioactivity, longer in-transit activity in the channels, and distinction between two nodes that lie close to each other and therefore appear as a single node on planar scintigraphy.

## Conclusions

Although this was a preliminary study, it is one of the few in the literature with more than 100 patients which has shown that the addition of SPECT/CT proved to be significant—76% in our patient cohort. Our findings demonstrate the inherent ability of SPECT/CT to provide superior images with increased nodal yield, more precise localization, a clearer distribution and drainage pattern, and a significant reduction in false extranodal hot spots observed on conventional planar imaging. Incorporating SPECT/CT can also deliver significant time efficiency gains, as it has the potential to replace static images (early and delayed) as well as the need for skin marking. The study has confirmed the advantages of SPECT/CT with respect to surgical planning and accuracy of lymph node detection, and we recommend its incorporated into the preoperative imaging protocol.
